# Clinicians’ Expectations of Web 2.0 as a Mechanism for Knowledge Transfer of Stroke Best Practices

**DOI:** 10.2196/jmir.2016

**Published:** 2012-09-13

**Authors:** Isabelle David, Lise Poissant, Annie Rochette

**Affiliations:** ^1^Institut de réadaptation Gingras-Lindsay-de-MontréalMontreal, QCCanada; ^2^School of RehabilitationFaculty of MedicineUniversity of MontrealMontreal, QCCanada; ^3^Centre de réadaptation Lucie-BruneauMontreal, QCCanada

**Keywords:** Qualitative research, health personnel, Internet, evidence-based practice, information dissemination

## Abstract

**Background:**

Health professionals are increasingly encouraged to adopt an evidence-based practice to ensure greater efficiency of their services. To promote this practice, several strategies exist: distribution of educational materials, local consensus processes, educational outreach visits, local opinion leaders, and reminders. Despite these strategies, gaps continue to be observed between practice and scientific evidence. Therefore, it is important to implement innovative knowledge transfer strategies that will change health professionals’ practices. Through its interactive capacities, Web 2.0 applications are worth exploring. As an example, virtual communities of practice have already begun to influence professional practice.

**Objective:**

This study was initially developed to help design a Web 2.0 platform for health professionals working with stroke patients. The aim was to gain a better understanding of professionals’ perceptions of Web 2.0 before the development of the platform.

**Methods:**

A qualitative study following a phenomenological approach was chosen. We conducted individual semi-structured interviews with clinicians and managers. Interview transcripts were subjected to a content analysis.

**Results:**

Twenty-four female clinicians and managers in Quebec, Canada, aged 28-66 participated. Most participants identified knowledge transfer as the most useful outcome of a Web 2.0 platform. Respondents also expressed their need for a user-friendly platform. Accessibility to a computer and the Internet, features of the Web 2.0 platform, user support, technology skills, and previous technological experience were found to influence perceived ease of use and usefulness. Our results show that the perceived lack of time of health professionals has an influence on perceived behavioral intention to use it despite favorable perception of the usefulness of the Web 2.0 platform.

**Conclusions:**

In conclusion, female health professionals in Quebec believe that Web 2.0 may be a useful mechanism for knowledge transfer. However, lack of time and lack of technological skills may limit their use of a future Web 2.0 platform. Further studies are required with other populations and in other regions to confirm these findings.

## Introduction

Gaps continue to be observed between professional practice and scientific evidence [1-3]. To address this situation, health professionals are increasingly encouraged to adopt an evidence-based practice. In Canada, a significant proportion of scientific evidence is not integrated into health care services offered to stroke patients [4-7]. The application of an evidence-based practice requires the implementation of resources and tools facilitating knowledge transfer and exchange between researchers and professionals.

According to the Cochrane Effective Practice and Organisation of Care (EPOC) taxonomy [8], 10 interventions are known to improve the practice of health care professionals: (1) distribution of educational materials, (2) educational meetings, (3) local consensus processes, (4) educational outreach visits, (5) local opinion leaders, (6) patient-mediated interventions, (7) audit and feedback, (8) reminders, (9) marketing, and (10) mass media. In the stroke field, 61 practice guidelines or educational materials exist worldwide according to a subcommittee of the World Stroke Organization [9]. One of those guidelines is published through the combined efforts of the Canadian Stroke Network and the Heart and Stroke Foundation of Canada. This guideline, the *Canadian Best Practice Recommendations for Stroke Care *[10], is published every 2 years (since 2006). Following this national initiative, each province has identified priorities and initiated an approach specific to their province.

In addition to educational materials, various knowledge transfer strategies have emerged in stroke care to reduce gaps between research and practice. Those strategies hardly fit in the EPOC taxonomy. As an example, the Montreal Stroke Network was created in 2002 independently of the national and provincial initiatives mentioned previously. This network has linked three communities of practice (prevention, acute care, and rehabilitation) and is composed of professionals working with stroke patients in Greater Montreal, a large urban city in Quebec, Canada. As presented in Poissant [11], the objective of the Montreal Stroke Network members is to improve the quality of professional practice in order to better meet the needs of stroke survivors across the continuum of care and in the community. The Montreal Stroke Network brings together a large variety of stakeholders (eg, managers, occupational therapists, nurses, physicians, speech-language pathologists, and social workers) working in different organizations (eg, hospitals, intensive functional rehabilitation units, and rehabilitation centers focusing on social integration). Communities of practice within the Montreal Stroke Network successfully developed and implemented several clinical projects.

In addition to the Montreal Stroke Network, initiatives such as Stroke Engine (www.strokengine.ca) [12] and Evidence-Based Review of Stroke Rehabilitation (www.ebrsr.com) [13] are available online for clinicians, managers, patients, and families to improve stroke care. Those strategies demonstrate that the Internet is well integrated into health care delivery. A study conducted among 243 health professionals (general practitioners, practice nurses, and practice managers) showed that 81% of them use the Internet for their work [14]. However, using the Internet for the sole purpose of accessing knowledge is unlikely to translate into practice change [15]. Conversely, accessing the Web to get involved in a community of practice is likely to lead to behavioral changes.

By implementing virtual communities of practice, communities can take advantage of new Internet applications. Web 2.0 is a “new generation of the Internet where interaction is important” [16]. Available interactive capacities within Web 2.0 facilitate information exchange among organizations independent of their geographic location. According to several authors [17-20], blogs, podcasts, and wikis are the most popular Web 2.0 applications. There are several other Web 2.0 applications (eg, virtual libraries and discussion forums). Several examples of virtual communities of practice are published in the health care literature [21,22]; however, little is known about the factors that will play a role in transforming a “face-to-face” community of practice into a virtual community of practice using Web 2.0 applications.

The main objective of this study is to document health professionals’ perception of a future Web 2.0 platform supporting knowledge transfer and implementation of stroke clinical practice guidelines. The secondary objective is to identify differences in perceptions between members of the Montreal Stroke Network and professionals outside this network possibly interested in joining it via a Web 2.0 platform.

## Methods

### Design

To understand professionals’ experiences regarding Web 2.0 being introduced into their practices, a phenomenological approach was chosen. Phenomenology, as described by Husserl in 1910, focuses on the essential structure of individual experiences. It allows for study of what is behind a phenomenon by giving the participant the opportunity to express freely and openly about the phenomenon. The analysis of the expressed content demonstrates the essence of what is perceived as inevitable following a rigorous process of reduction [23].

### Population

Clinicians and managers working with stroke patients in a health organization of Quebec were asked to participate in the study. To compare the needs of Montreal Stroke Network members with those outside this network, 3 groups were targeted. The first group consisted of members of the Montreal Stroke Network. The second group was composed of health professionals who were considered members of the network, but who remained inactive over the previous 2 years. Lastly, the third group gathered health professionals who did not know about the network and who were working in remote areas. The target sample size was 20 people to have a holistic representation of each profession in every stroke care continuum phase (ie, acute care, intensive functional rehabilitation, and community reintegration).

### Recruitment

We used a purposive sampling strategy to ensure adequate representativeness of groups. Therefore, we used the membership list of the Montreal Stroke Network to identify and contact participants for the first 2 groups (active and inactive Montreal Stroke Network members). We contacted research coordinators of rehabilitation centers and acute care hospitals geographically distant from the city of Montreal and asked them to identify health professionals who could potentially participate in the study. Potential participants were contacted by email or phone.

### Data Collection Method

We conducted individual semi-structured interviews with each participant from January to July 2010. Semi-structured interviews were preferred as the data collection method because they are consistent with a phenomenological approach. The interview guide was developed on the basis of available scientific literature on Web 2.0 capabilities, communities of practice, and technology acceptance. A first version of the interview guide was pretested with a health professional to verify interview length and clarity of the questions. Revisions were made by the research team. The interview guide was structured as follows. First, participants explained their job particulars, then they discussed their perception and their needs regarding a Web 2.0 platform more specifically. Issues related to strategies used to share knowledge, benefits of a community of practice, and technology use were also discussed. All interviews were conducted by the same member of the research team (ID). Because the interviews were conducted prior to the development of the platform, key Web 2.0 definitions were provided to participants to ensure standardized comprehension of the concepts. The length of interviews varied from 40 to 75 minutes. The interviews were conducted in French or in English at the participant’s convenience. A reflective research diary was used for gathering information about contextualization and interpretation of data. Most interviews were face-to-face, but 5 interviews were conducted by telephone due to geographical distance. Written consent was obtained from all participants.

### Data Analysis

The audio content of each interview was transcribed. A member of the research team (ID) verified the accuracy of the transcripts and conducted the content analysis. Two other research team members (LP and AR) reviewed and discussed the codes to make sure they had face validity. First, codes were assigned to units of meaning and they were grouped into themes. Next, the research team identified links between identified codes. Only codes that were considered essential to answer the research question were kept. Qualitative data analysis software (QDA Miner 3.2.6) was used for data management and to support a systematic analytic approach by the research team. The research diary was used to refine the results and keep track of decisions made during the analysis.

### Ethics Approval

This study was approved by the Ethics Committee of the Centre for Interdisciplinary Research in Rehabilitation of Greater Montreal.

## Results

### Sample Description

Twenty-four health professionals agreed to be interviewed: 9 were active Montreal Stroke Network members (“Active members”), 9 were inactive Montreal Stroke Network members (“Inactive members”), and 6 were in the remote areas group (“Remote members”) (see Table 1). Six people didn’t answer the invitation and 1 person refused. The distribution of stroke patients seen by health professionals within each group was similar. The interviews were conducted in English with 2 participants (participants A3 and I9). All participants were women with an average age of 45 years (SD 9.64). Mean professional experience was 18 years (SD 8.75). Nearly two-thirds had access to an individual computer. Participants in the Active members group were significantly older (mean 51 years, range 39-64, *P *= .02) than participants in the Remote members group (mean 38 years, range 28-46 years).

**Table 1:** Characteristics of participants.

		Participant	Age (years)	Profession^a^	Professional experience (years)	Experience with stroke patient (years)	Clinical time (%)	Workplace^b^	Ratio (computer/ individual)	
**Active members**									
		A1	48	OT	25	19	50	IFRU	1:10
		A2	48	PT	26	22	100	IFRU	3:10
		A3	66	SLP	30	25	66	Acute care	1:1
		A4	62	Program manager	18	4	0	Community reintegration	1:1
		A5	40	Nurse	18	10	100	IFRU	1:1
		A6	39	Clinical-administrative manager	6	1	0	IFRU	1:1
		A7	47	Clinical nurse specialist	24	20	90	Acute care	1:1
		A8	49	OT	25	20	98	Acute care	3:5
		A9	64	Neurologist	32	20	50	Acute care	1:1
**Inactive members**									
		I1	39	OT	15	15	100	Acute care	3:5
		I2	44	OT	21	20	100	Health and social services center	1:2
		I3	46	Consultant nurse	23	11	0	Acute care	1:1
		I5	47	PT	24	5	100	Acute care	3:11
		I6	40	PT	17	10	100	Specialized acute care	1:6
		I7	55	SW	33	25	100	Acute care	1:1
		I8	33	Nurse	9	5	80	Acute care	1:1
		I9	48	Neurologist	14	14	85	Acute care	1:1
**Remote members**									
		R1	28	PT	3.5	3.5	100	Acute care	1:5
		R2	30	OT	7	2	100	Community reintegration	1:1
		R3	40	Clinical nurse specialist	10	2	100	Community reintegration	1:1
		R4	46	Neuro-psychologist	25	11	100	IFRU	2:5
		R5	46	Clinical coordinator	20	9	0	IFRU and community reintegration	1:1
		R6	37	Program manager	5	5	0	IFRU and community reintegration	1:1

^a ^OT: occupational therapist; PT: physical therapist; SLP: speech-language pathologist; SW: social worker

^b ^IFRU: intensive functional rehabilitation unit

### Results of Objective 1: Health Professionals’ Perception

Four themes documenting perception of Web 2.0 emerged from the interviews: (1) influence of external variables, (2) perceived usefulness, (3) perceived ease of use, and (4) time availability. External variables were associated to accessibility, system features, user support, technologic skills, tool experience, and profession. Those variables had an impact on perceived usefulness and perceived ease of use. Perceived usefulness was characterized with concepts referring to knowledge transfer, quality of care, and efficiency. Perceived ease of use was associated with 2 codes: user-friendliness and timely access to information. The results indicated that participant’s profession, one of the external variables, influences the time availability for technology utilization. Finally, the combination of perceived usefulness and perceived ease of use was creating the behavioral intention to use the technology. The results suggested that time availability influences system utilization in addition to behavioral intention. Examples of quotes supporting these results are presented in Table 2.

**Table 2:** Themes, codes, definitions, and sample statements.

Theme	Code	Definition	Statement
External variables	Accessibility	Computers performance and availability; quality of the Internet connection at work	“We have old computers” (participant I1)
	System features	Characteristics of Web 2.0 applications and exchanges	See Table 3
	User support	Informatics support available at work	“We have an informatics department here. Whenever something doesn’t work, we call them and they can fix it rapidly normally” (participant A2)
	Technological skills	Level of skills to use computer and the Internet	“I am skilled to do what I have to do” (participant A7)
	Tool experience	Previous emotional experience with a Web 2.0 platform	“I could spend my whole day here. I had to stop, because it could take my whole day, I’m very interested, it could take up my whole day” (participant A3)
Perceived usefulness	Facilitates knowledge transfer	Opportunity to learn and stay up-to-date through exchanges with other members of a Web 2.0 platform	“I think it might be interesting, the opening, to have contact with other people working in the same field. Especially if it’s interactive, I think that’s good too, to have access to information with an easier way than right now” (participant A1); “To have access to what is done in other organizations” (participant I3); and “To seek the others’ expertise.” (participant I6)
	Increased quality of care	Opportunity to make changes to improve care through a Web 2.0 platform	“It can get answers to people...which may be useful in their practice” (participant A4)
	Allows tasks to be accomplished more quickly	Opportunity to decrease time spent to search information or to do other tasks through a Web 2.0 platform	“Why reinvent something when it already exists?” (participant R5)
	Perceived uselessness	Personal and organizational resistance to change	“From a management point of view, I have a concern with how clinicians will use this tool and how much time they will spend on it” (participant A6)
Perceived ease of use	User-friendly	Intuitive learning of a Web 2.0 platform	“If it’s complicated, it might unmotivate me” (participant I7)
	Timely access to information	Optimizing the time fit between an informational need and its answer through a Web 2.0 platform	“If I have a problem, I need a quick response” (participant A7)
Time availability	-	Available time to learn about best practices and to search on the Internet	“Nobody has the time to do that” (participant I8)
Behavioral intention to use	Positive	Expected platform use	“By using it, if everything is going well, I will use it more and more often” (participant R4)
	Negative	Unexpected platform use	“I don’t think I will go on it (participant the platform) every day” (participant I8)

#### External Variables

Within external variables influencing perceived usefulness and perceived ease of use, two were related to the technology (accessibility and features of the Web 2.0 platform), three were related to users (technologic skills, tool experience, and profession), and one was related to technological support provided to users. Accessibility to computers appeared to be influenced by the type of profession. Indeed, the ratio of computers to professionals could be as low as 1:10 for occupational therapists and physical therapists (see Table 1). In comparison, neurologists, speech-language pathologists, clinical nurse specialists, and workers with a management role (program manager or clinical coordinator) each had a personal computer (1:1 ratio). Many participants also reported having access to poorly performing computers and slow Internet connections at work.

Because of its complexity and in light of our interview process, 5 subcodes were attributed to the features of the Web 2.0 platform. These subcodes were identification, Web 2.0 applications, animation, look and feel, and membership fees (see Table 3).

**Table 3:** System features of subcodes.

Subcode	Definition	Statement
Identification	Password to access the platform. Once connected, personal information is revealed.	“Personally, I wouldn’t have trouble identifying myself: where I’m from, what is my profession, where I work, my name...But some people might be less comfortable with this” (participant A5)
Web 2.0 applications	Perceived relevance of Web 2.0 applications (eg, blogs, podcast, and forums). Concerns about the quality, the relevance, and the variety of exchanges.	“I want quality answers” (participant R3)
Animation	Designated person to stimulate and organize exchanges.	“Someone will monitor that? Someone will manage that?” (participant A1)
Look and feel	Platform visual.	“If it is attractive, it is for sure an advantage” (participant I5)
Membership fees	Money to pay for the membership.	“When it’s free, it’s evident that I will try it for a time period” (participant I3)

Identification referred to the log-in process to start using the Web 2.0 platform and to the identification of participants once on the platform. Given the general abundance of passwords individuals have to deal with, participants perceived a secured log-in process as a barrier. Once on the platform, the respondents’ perception of the importance of identifying themselves by their name, profession, and workplace was divided. Half of the participants felt that names should appear, one-quarter believed that people should have the choice to identify themselves or not, and one-quarter did not want to identify themselves at all. In terms of accessing information about a members’ profession, more than half agreed with it being displayed, and no one completely disagreed. The remaining respondents were ambivalent or would give members the choice to display it or not. Most participants agreed that anonymity could influence the assessment of the quality of the information.

Professionals didn’t express issues regarding patient confidentiality within virtual exchanges. It seemed like they were fully aware that written communication within a Web platform must ensure confidentiality and respect ethics rules. However, some of them were enthusiastic and some were reluctant about patients’ prospective access to a section of the platform.

High variability was seen among participants about the most relevant Web 2.0 applications for professional practice. Nevertheless, some preferences were observed. Access to videos and a list of pertinent websites were preferred to podcasts and wikis. Two other potentially useful applications were virtual libraries and calendars because they facilitate access to documents and events that shared by colleagues. Email notifications were appealing to professionals. Although some were apprehensive about the potentially large volume of notifications, others saw notifications as a means to save time. Asynchronous exchanges on a Web 2.0 platform emerged as more applicable to professional practice and time management than synchronous exchanges. A preference was also noted in favor of discussion forums rather than blogs. Professionals gave added value to forums that were perceived as more time efficient because of the way the threads are organized. Participants mentioned that the rating of forum threads would be more useful than knowing the number of times the thread was read. Respondents believed that threads must be relevant and contain reliable and quality information.

participant I6 “It is easy to write things in the forums, but...there are those who get carried away and put too much.”

participant I8 In fact, some professionals only wanted to access expert opinions: “The problem that I see with forums is that you have to read the opinion of everybody...All I want is the expert.”

In addition to this concern for quality, discussion or information sharing should give professionals the feeling that they are learning while staying focused on clinical practice.

participant A3 “If the level of discussions weren’t interesting, I would eventually stop. Or if the level was, you know...the level was interesting and the questions were serious, then I’d go.”

participant R5 “Theoretical and scientific information is easy to access. I think that it is more the things in day-to-day life, information in the field that are...that are less accessible.”

An expressed challenge was the importance of having varied topics to interest all members regardless of their profession and workplace. Finally, the update frequency was an important feature to consider. The need to access up-to-date information integrating evidence-based knowledge and innovative and yet-to-be-proven knowledge was frequently mentioned during interviews.

participant I1 “We do not have the time to go use [a Web 2.0 platform], because that changes all the time.”

participant I6 “There are sites that you consult and you return to see them, and realize that it’s been a year and they have not been updated.”

All respondents thought facilitators should oversee the knowledge management process to increase the credibility of a Web 2.0 platform. Almost one-third of participants said that the “look and feel” would have an impact on their use of the platform. Lastly, according to some respondents, membership fees would constitute a barrier.

For most respondents, an efficient information technology (IT) department was already in place in their work environment. The relevance of having additional support for a Web 2.0 platform did not come out in the interviews.

According to respondents’ self-assessment of their technology skills, most professionals working in remote areas had good computer skills. Active and Inactive members said they had limited skills.

Some participants mentioned they had a pleasant previous experience with a blog or a forum related to their job. Participant A3 claimed becoming over-addicted to this type of tool in the past, and was forced to stop using it. Participant I3 claimed to have a growing interest in forums or blogs, but was still underusing Web 2.0 platforms. Participant I4 occasionally used a discussion forum. Some other respondents (participants R2, R4, and R5) used these types of platforms outside their professional life. Others did not report any previous experience.

#### Perceived Usefulness

Participants identified three main uses of Web 2.0. The primary use mentioned by the majority of respondents was knowledge transfer. According to them, through interactions and discussions, Web 2.0 platforms offer opportunities to learn and remain up-to-date. It is also a means to facilitate information gathering.

Respondents perceived that young health professionals and professionals working in remote areas would be most likely to benefit from knowledge exchanges that would take place on the Web 2.0 platform. Some participants (participants I3, I8, I9, and R6) mentioned that Web 2.0 applications would be more useful for patients than professionals.

For several health professionals, a Web 2.0 platform would offer an opportunity to link research evidence to clinical practice.

participant I4 “It could be grouped by things that are...with evidence-based...and those looking for the clinical aspect...we make sure to be on the right track than to use trial or error.”

A second use of a Web 2.0 platform was linked to its capacity to increase quality of care. Participants felt that knowledge transfer and exchanges via the Web 2.0 platform would eventually increase the quality of work through behavior changes related to service delivery and care standardization.

However, concerns were expressed about avoiding duplication of resources.

participant R1 “It would be good if the website gathers the information...instead of spreading it.”

The third use of the platform was to allow professionals to accomplish tasks more efficiently. A Web 2.0 platform could reduce time spent searching for information by expanding their sources of information through a larger network of colleagues. It might also be useful for health care providers because it pools efforts made in other workplaces. Respondents also felt the Web 2.0 platform could reduce travel time for meetings.

participant A1 “I can have the same question as someone else, and it’s already there. It saves a search that is regularly done. I find that is practical.”

participant I3 “We may lose less time to build things, but just adapt them to our setting.”

Elements describing the participants’ resistance to the introduction of Web 2.0 into their practices cannot be ignored. This resistance was noted more frequently among managers and late-career professionals (eg, participants A6, I7, and R6). For example, a manager was afraid that employees would use that kind of tool for entertainment instead of work. A lack of organizational recognition was also identified as a barrier to the use of the platform.

participant I6 “It is not just the time to treat patients, we also need time to...for other things. It is less recognized in my organization. It’s really, really focused on how many patients you have seen.”

Some participants considered that other means, such as face-to-face discussions or email, are sufficient for their knowledge transfer needs.

participant I8 “If I have a question, I will use my email and will send it to someone.”

Finally, another description of perceived uselessness referred to the inequities between academic and non-academic organizations. Here is how a professional from a non-academic organization expresses her view: “Budget is not the same, things are not the same, so it’s not necessarily easy and you often feel incompetent when you compare your practices” (participant I1). This participant negatively perceived Web 2.0 exchanges with professionals working in academic organizations.

#### Perceived Ease of Use

To encourage people to use a Web 2.0 platform, it has to be as user-friendly as possible: “If it is easy to use, we will use it more” (participant R2). Providing clear and well-organized information was seen as a key factor to limit learning required to use the platform and to save time.

participant A2 “That things are well organized and we do not get lost in finding information, it is also good to encourage us to consult more often.”

Being able to access the information at any time, in any place, was seen as a major advantage of Web 2.0 platforms over face-to-face or phone exchanges. To meet respondents’ expectations, pages on a Web 2.0 platform must download quickly, and answers to questions should be made available in a timely fashion.

participant I7 “If I have a problem, I have to quickly have access to get a fast answer. Because I will not go back three times.”

#### Time Availability

Almost all respondents mentioned they lacked time to read about scientific evidence and to use tools such as Web 2.0 platforms. Professionals explained that these tasks were not necessarily valued in workplaces. Individuals who did not mention this barrier were managers or clinical coordinators (participants A4 and R5).

#### Behavioral Intention to Use

The vast majority of participants expressed their intention to use the platform. Utilization would be gradual and would vary depending on members’ needs (participants I1, I2, I5, R3, and R4).

participant R4 “The better it goes, the more we will return, and it becomes somewhat automatic.”

A realistic frequency of use was defined as once per week. Respondents expressed an “intention to use” ranging from 2 to 15 minutes per visit. Professionals expressed a low tendency to interact actively on the platform. They were more likely to access or view information than to contribute or add new information. This phenomenon was also expressed by a respondent who already uses Web 2.0 in her practice. Some participants (participants I8, R1, and R4) expressed little or no intention to use the Web 2.0 platform. According to participant I8 (33 years), nobody has the time to use a Web 2.0 platform as part of his or her job. Participants R1 (28 years) and R4 (46 years) do not think they will use this type of platform.

### Results of Objective 2: Group Differences

Some differences were seen between the 3 groups studied. First, Remote members seemed to have better access to computers. Moreover, this group seemed to have access to a larger network of colleagues outside their organization. Indeed, Active and Inactive members interacted less often with colleagues outside their organization compared with participants working in remote areas. Lastly, the possibility of increasing quality of work was not mentioned by the group working in remote areas, although half of the respondents in the other 2 groups expressed it. No difference concerning available time was observed.

## Discussion

**Figure 1:**
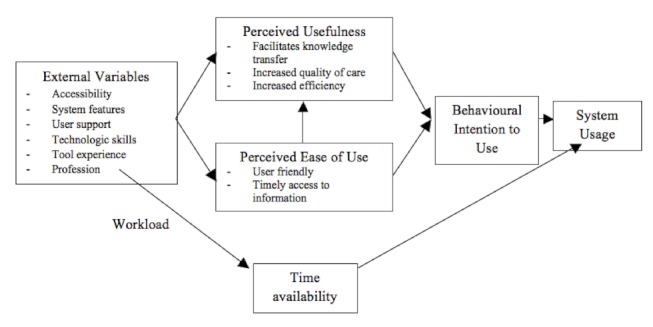
Modified Technology Acceptance Model (TAM) reflecting perceived factors influencing Web 2.0 adoption by health professionals.

The main objective of this study was to document professionals’ perception regarding the use of Web 2.0 in their clinical practices. Four out of 5 themes that emerged from our content analysis correspond to the Technology Acceptance Model (TAM) concepts (see Figure 1) [24,25]. Only, the “time availability” theme did not correspond to this model. This conceptual model was developed by Davis in 1980 to better understand why individuals accept or reject technology [24,25]. Although it is not specific to the health care system, it has been widely used to understand IT adoption by health professionals [26]. The TAM has been used with various professionals (eg, nurses [27,28], physical therapists [29], and occupational therapists [30]) using different types of information and communication technologies.

Like Van Schaik [29], our study demonstrates that technology is perceived as a support to evidence-based practice. More specifically, knowledge transfer is identified as the main use of the Web 2.0 platform. In the health care field, this is a major issue [31,32]. According to the Canadian Institutes of Health Research, knowledge transfer is “a dynamic and iterative process that includes the synthesis, dissemination, exchange, and ethically sound application of knowledge to improve the health of Canadians, provide more effective health services and products, and strengthen the health care system” [33]. Knowledge transfer should promote exchanges among patients, health professionals, managers, and researchers. In our study, professionals mainly expressed their need to share clinical experiences among themselves. It is interesting to observe that information searching based on collaborative networks is part of Eysenbach’s Medicine 2.0 definition [34]. Eysenbach named this concept “apomediation.” But it is important to remember when new knowledge is emerging, clinical experience, or tacit knowledge, is as important as explicit knowledge [35].

For respondents, ease of use of a Web 2.0 platform translates into a platform that does not require prior training (ie, is intuitive). This is important in the current health care system in Quebec where resources dedicated to training are scarce and time availability for integrating new knowledge is limited. As observed by Spallek [36], up-to-date and timely information are foundational elements of an emergent community of practice. The use of a Web 2.0 platform to obtain information in a timely fashion seems especially important to professionals working in remote areas despite their access to a large network of colleagues. Active members, working in urban regions, are probably part of larger teams and their organizations may have more human and financial resources (eg, documentation center, affiliated researchers, and clinical coordinator). This may reflect that professionals still tend to consult their immediate peers when facing complex situations, something that professionals in remote areas cannot do because of limited access to on-site peers.

Despite high motivation from respondents to use a Web 2.0 platform that would be easy to use and would offer added value to their practice, several barriers remain. Time was the most often reported barrier. This is consistent with the results reported in other studies that looked at Internet or technology use among various professionals (eg, physicians, nurses, managers, and physical therapists) [14,29,37,38]. This lack of time is also discussed in studies about professionals’ involvement in virtual communities of practice (eg, medical imaging administrators and emergency clinicians) [21,22]. Having available time is absolutely essential to participate in a community. This time availability is noted by Wenger et al [39] as being an important element to consider in the early stages of community development. Indeed, time is required before community members can see the added value it gives to their work. This reality is a challenge because respondents expressed their need for rapid answers. This situation reveals a contradiction where professionals will have to make coherent choices. Although they expect to save time by using a Web 2.0 platform to answer their knowledge needs, they will need to invest time to avoid disruption in the flow of information that will be conveyed by the platform. Organizations will need to address this issue to optimize professionals’ time and further research will need to be conducted to assess the utilization of a Web 2.0 platform given the short amount of time professionals are willing to spend on such knowledge transfer tools.

During content analysis, classification of external variables influencing perceived usefulness and ease of use was particularly challenging. These variables cover a large spectrum of fields and characteristics with no clear pattern in the selection of external variables across studies [26]. Despite this, several studies have mentioned the low competency of health professionals in using tools available on the Web (eg, databases, virtual communities of practice, and other online information) [14,22,37,40]. Our study supports this observation in that a significant proportion (37.5%) of respondents estimated they had low technological skill. Information technology training should be part of university programs and continuing education sessions for health professionals to enhance their professional skills and encourage behavior changes. According to cyberpsychology, technological skills are correlated with age [41,42]. This relationship has not been identified in our results because the participants who had no intention of using a Web 2.0 platform were between 33 and 46 years. Moreover, no relationship was identified between expressed intention to use and technological skills.

Although it is not shown in our adaptation of the TAM, some respondents perceived a Web 2.0 platform as having little or no use for knowledge transfer. They did not perceive the need to introduce a new knowledge transfer strategy illustrating the existence of other means of knowledge transfer, whereas others reported lack of support from the organization, putting more emphasis on the number of patients treated, two concepts defined by Paré [43] as “vision clarity” and “organizational flexibility.”

When comparing the 3 subgroups, very little difference is observed. Professionals working in remote areas may represent a group who would easily accept the introduction of a Web 2.0 platform into their practices because they seem to have better access to computers. However, according to these results, they were less likely to perceive this new knowledge transfer strategy as a useful one because of already well-established networks outside their workplace. Moreover, the fact that members of the Montreal Stroke Network and people within the periphery of the Montreal Stroke Network interact less with colleagues outside their organization could possibly explain why only 2 participants from the Montreal Stroke Network mentioned that it is important that the Web 2.0 platform meet their information needs at the right time, whereas this need was reported by almost all respondents from the other 2 groups.

### Limitations

Our study has some limitations. First, all respondents were women. However, according to the 2006 Canadian Census [44], 4 out of 5 health workers are women (80%). The fact that only 4 participants worked strictly with stroke patients could be seen as a limitation since the future Web 2.0 platform is intended to be focused on stroke care. Again, our study population is representative of the current organization of care, where health professionals must often deal with multiple clienteles [45]. Another limitation refers to empirical saturation. Despite the number of interviews (n = 24), empirical saturation in each group was probably not reached. We used a purposive sampling approach to recruit our participants when participants should usually be chosen based on the intensity of their experience of the phenomenon under the phenomenological approach. Respecting this condition would have limited our capacity to recruit since Web 2.0 applications are still emerging in the health care field. To compensate for this situation, we asked respondents about their level of skills with technology and we had a variety at each level. The interview as a data collection method possibly created a social desirability bias related to the expressed intention to use the Web 2.0 platform. Further studies are needed to take into consideration the organization and system levels in addition to the individual level as recommended by Karsh [46]. Lau [47] already demonstrated that health policy makers have to be involved in promotion of Web 2.0 utilization. Lastly, it is important to be aware that in qualitative research results may be transferable, but are not necessarily generalizable.

### Conclusion

In this study, we aimed to understand professionals’ perceptions and needs regarding the introduction of a future Web 2.0 platform into their practices. Previous studies have shown that a positive attitude is often associated with a high level of technology acceptance and adoption [48,49]. Our results reveal that professionals consider Web 2.0 to be very useful for knowledge transfer. However, lack of time and lack of technological skills are limitations to their future use of this technology. The introduction of Web 2.0 platforms undoubtedly requires a change in work habits. Professionals still seem to be inclined to use general search engines (eg, Google) to meet their information needs, whereas sites more specific to their profession could allow them to access more relevant information. Eventually, it might be interesting to investigate patients’ perceptions of Web 2.0 platforms technology because this technology may be of interest to them (as stated by some of the respondents). It also might be interesting to explore health professional openness to introduce Web-based exchanges with stroke patients such as Nordqvist [50] addressed it for health professionals and diabetes patients.

## Abbreviations

EPOC: Cochrane Effective Practice and Organisation of Care
QDA: qualitative data analysis
TAM: Technology Acceptance Model
